# IVCA in a Boy with Multilocular Renal Cyst as a Risk Factor for Deep Vein Thrombosis

**DOI:** 10.34763/jmotherandchild.20242801.d-24-00045

**Published:** 2025-03-25

**Authors:** Agnieszka Szmigielska, Piotr Skrzypczyk, Michał Szyszka, Magdalena Bukowska, Malwina Wojtas, Aleksandra Jakimów-Kostrzewa

**Affiliations:** Department of Pediatrics and Nephrology, Medical University of Warsaw; Extracurricular Medical Students Scientific Association, Department of Pediatrics and Nephrology, Medical University of Warsaw; Department of Pediatric Radiology, Medical University of Warsaw

**Keywords:** agenesis, inferior vena cava, KILT syndrome, renal cyst, deep vein thrombosis

## Abstract

**Introduction:**

The triad of symptoms: renal defects, congenital inferior vena cava agenesis (IVCA) and deep vein thrombosis of the lower limbs make up the KILT syndrome (kidney and IVC abnormalities with leg thrombosis).

**Case report:**

A 17-year-old boy complained of periodic abdominal pain. Abdominal ultrasonography revealed a multilocular cyst in the right kidney. Physical examination showed no abnormalities, and his blood pressure was 120/80mmHg. Abdominal ultrasonography showed a cyst measuring 36×30×25mm in the right kidney hilum. Computed tomography did not show the hepatic and suprarenal sections of the inferior vena cava. Numerous varicose-dilated collateral vessels, including renal venous vessels, were found in the right kidney hilum. The collateral vessels in the tomography matched the described in the ultrasound renal cyst. MRI confirmed IVCA with no other additional vascular abnormalities. Due to the risk of deep vein thrombosis of the lower limbs, non-pharmacological antithrombotic prophylaxis was recommended.

**Conclusions:**

Early detection of inferior vena cava agenesis allows for the reduction of the risk of dangerous thrombotic complications.

## Introduction

The incidence of single renal cysts is estimated to be approximately 10.7%. The primary risk factor for cyst formation is age. In the second decade of life, kidney cysts are found in 2.38% of patients, whereas the frequency increases to 35.29% in individuals aged 70 years and older. Simple renal cysts are more common in boys than in girls [1]. Renal malformations are often accompanied by other anomalies, including vascular defects. Inferior vena cava (IVC) malformations occur in about 0.3–0.5% of healthy individuals, but their prevalence increases to 0.6–2% in patients with vascular incidents [2]. Inferior vena cava agenesis (IVCA) is a rare condition, affecting 0.0005–1% of the general population [3].

IVC anomalies are recognized as an independent risk factor for lower extremity deep vein thrombosis (DVT). Among young patients with DVT, approximately 5% are found to have IVC anomalies [4]. IVCA is an important developmental anomaly that can lead to DVT; therefore, early detection is essential for preventing this potentially serious complication. The coexistence of IVCA, renal defects, and thrombosis characterizes KILT syndrome, first described by Van Veen in 2002 [5].

## Case Report

**Figure 1.**
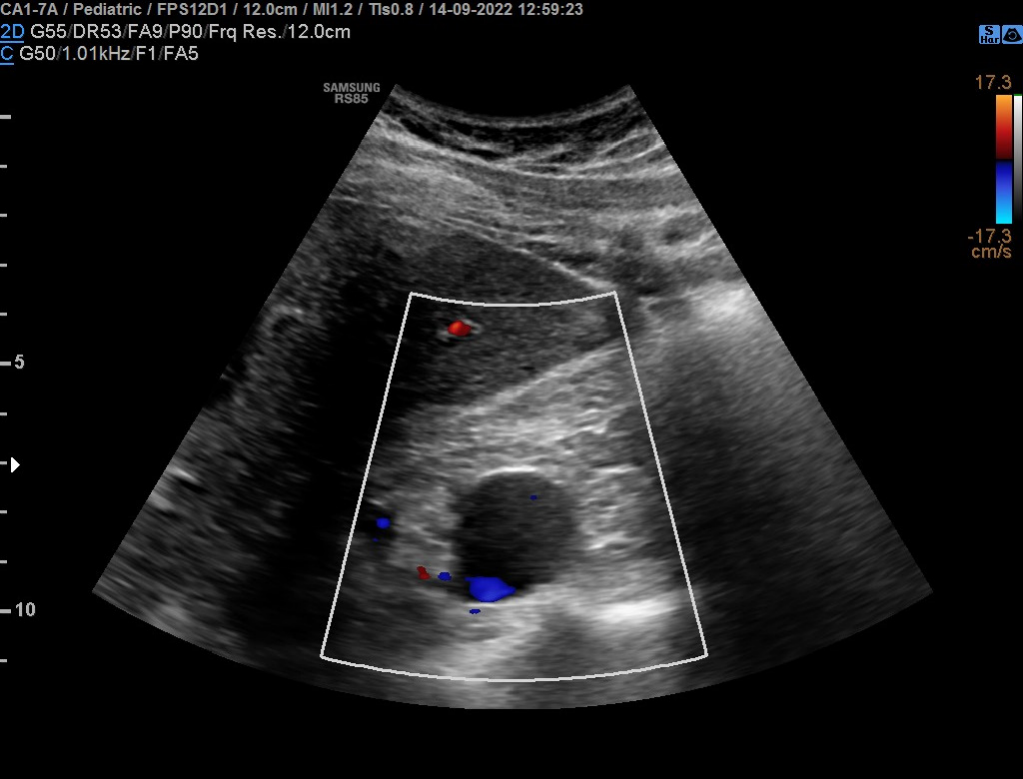
Abdominal ultrasonography shows multicellular cyst in the hilar region of the right kidney.

**Figure 2.**
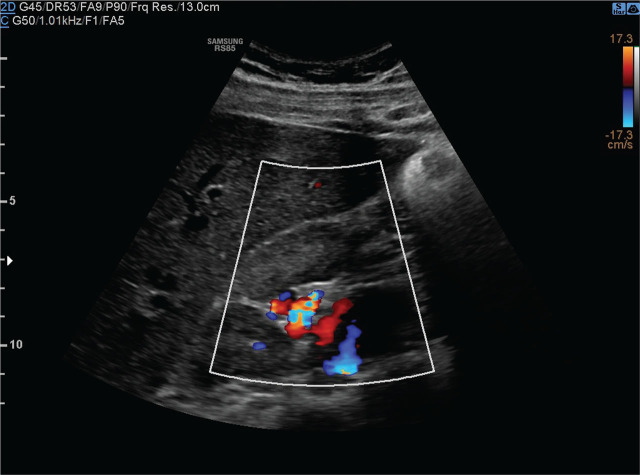
Abdominal ultrasound with power doppler shows blood flow within the right kidney cyst - peripheral circulation.

**Figure 3.**
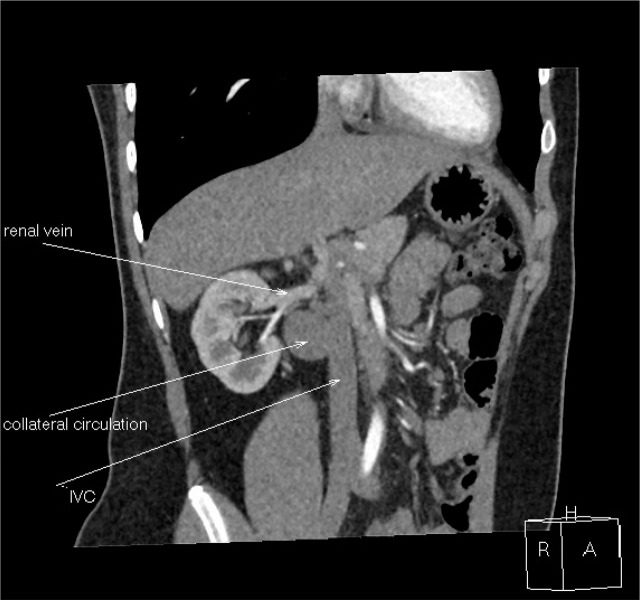
CT scan of abdomen: Lack of hepatic and suprarenal IVC, venous outflow leads through vena azygos and hemiazygos.

A 17-year-old boy was admitted to the hospital due to intermittent abdominal pain that had persisted for six months. On admission, his general condition was good. Physical examination revealed no abnormalities, and his blood pressure was 120/80 mmHg. Laboratory tests, including inflammatory markers, liver and kidney function parameters, coagulation profile, and urinalysis, were all within normal limits. Abdominal ultrasound showed that both kidneys were of normal size (110–112 mm). However, a large multilocular cyst measuring 36 × 30 × 25 mm was identified near the hilum of the right kidney (Fig. 1). During the examination, the cyst’s volume was noted to change with the patient’s breathing pattern. Doppler ultrasound demonstrated a flow spectrum consistent with venous flow (Fig. 2). Computed tomography (CT) did not visualize the hepatic or suprarenal segments of the IVC. Venous outflow was redirected through the azygos and hemiazygos veins (Fig. 3). Collateral circulation, including numerous renal veins near the right renal hilum, was identified, corresponding to the ultrasound findings. Magnetic resonance imaging (MRI) confirmed the absence of the IVC at the level of the kidneys and demonstrated extensive collateral circulation in the right hilar region, measuring 31 × 30 mm. Given the increased risk of DVT, non-pharmacological thromboprophylaxis was recommended. This included elevating the legs above heart level during rest, maintaining adequate hydration, engaging in regular physical activity, avoiding prolonged immobility, and using compression stockings during high-risk activities, such as long-haul travel.

## Discussion

IVC agenesis (IVCA) is a rare vascular malformation with a complex aetiology. Abnormal embryogenesis during early pregnancy (4–8 weeks) can lead to impaired venous outflow from the right metanephros, resulting in abnormal development of the right kidney [6]. Some authors suggest that IVCA may also arise from perinatal or intrauterine thrombosis [7]. IVC anomalies frequently accompany renal defects, such as agenesis, hypoplasia, or aplasia, and often involve the right kidney. Patients with IVCA may be referred to a nephrologist for suspected renal malformations based on abnormal ultrasound findings. Collateral circulation and thrombotic complications can lead to renal dysplasia at different ages [8]. Cases involving the left kidney typically present with hypoplasia, renal artery stenosis, and hypertension [9].

IVCA often remains asymptomatic and is discovered incidentally during imaging for unrelated symptoms, such as infections [10]. When symptoms do occur, they commonly include abdominal, sacral, or groin pain. In older children, varicose veins in the lower extremities or abdominal wall may be evident [11]. In the present case, the 17-year-old boy’s laboratory tests did not identify the cause of his abdominal pain. Ultrasound revealed a fluid-filled space in the right renal hilum, suggesting a multilocular cyst. The change in the cyst’s volume with breathing indicated vascular anomalies and possible collateral circulation.

Doppler ultrasound is the preferred initial imaging study when IVCA is suspected. However, IVCA may not always be detectable via Doppler. CT angiography or MRI should be used for confirmation [12].

DVT is often the first clinical sign of IVCA. In children, the incidence of lower extremity venous thrombosis is approximately 1 in 10,000. Risk factors for thrombosis include thrombophilias (congenital or acquired), immobilization, and malignancy [13]. When a child develops DVT, it is crucial to exclude IVCA and other common risk factors. Limited collateral circulation in IVCA patients can lead to venous stasis, significantly increasing the risk of thrombosis. Pulmonary embolism is uncommon in IVCA patients with DVT.

In the presented case, IVCA was detected before the onset of DVT complications. Pharmacological treatment was not required; however, non-pharmacological measures were recommended including physical activity, the use of compression stockings, and avoiding smoking. Patients at high risk for DVT should be educated on recognizing complications, such as pulmonary embolism (e.g., shortness of breath or chest pain), and the importance of seeking urgent medical care. Non-pharmacological strategies play a crucial role alongside medical therapy in DVT management.

Treatment of DVT in IVCA patients typically involves low-molecular-weight heparins (LMWH). The REVIVE trial demonstrated the superior efficacy of LMWH compared to unfractionated heparins, followed by oral anticoagulation [14]. In select cases, anticoagulant therapy should be considered for DVT prevention.

## Conclusions

When diagnosing renal cysts, the possibility of IVCA and collateral circulation in the renal hilum should be considered. In children presenting with lower extremity venous thrombosis, comprehensive imaging studies should be performed to exclude IVCA, in addition to testing for thrombophilia.
